# Serum biomarkers for predicting Crohn’s disease activity: The role of bilirubin, uric acid, and the C-reactive protein/albumin ratio

**DOI:** 10.1371/journal.pone.0333855

**Published:** 2026-03-04

**Authors:** Ting Xu, Tingting Yao, Yating Chen, Fan Xu, Chunxia Lu

**Affiliations:** Department of Gastroenterology, the Second Affiliated Hospital of Anhui Medical University, Hefei, Anhui, China; University of Montenegro-Faculty of Medicine, MONTENEGRO

## Abstract

**Background:**

Crohn’s disease is a chronic, progressive inflammatory condition of the gastrointestinal tract that requires long-term assessment of disease activity. There is a growing need for convenient serum biomarkers to reduce the need for invasive endoscopic evaluations. Our study aims to explore the predictive value of serum biomarkers, specifically total bilirubin, uric acid, and the C-reactive protein/albumin ratio, for disease activity in Crohn’s disease.

**Methods:**

We conducted a retrospective study at the Second Hospital of Anhui Medical University (China), consisting of 170 patients with Crohn’s disease and 100 healthy controls. Clinical characteristics and laboratory biomarkers were collected and analyzed. Among the patients, 77 active Crohn’s disease patients who had complete follow-up data were included in a longitudinal analysis to assess biomarker dynamics.

**Results:**

Compared to healthy controls, Crohn’s disease patients exhibited lower bilirubin and albumin levels, but higher C-reactive protein and C-reactive protein/albumin ratio, trends that intensified with disease progression. Following therapy, albumin, C-reactive protein and C-reactive protein/albumin ratio changed significantly in both remission and active groups, whereas a significant increase in total bilirubin was exclusive to the remission group. Receiver operating characteristic analysis indicated that C-reactive protein/albumin ratio had the highest discriminatory power for disease activity (area under the curve [AUC]= 0.903), outperforming C-reactive protein alone (AUC = 0.894), albumin (AUC = 0.719) and total bilirubin (AUC = 0.648). Regarding uric acid, no significant associations were identified overall, apart from a single weak correlation in Spearman’s analysis.

**Conclusion:**

Our findings support a potential serological remission hypothesis for Crohn’s disease, characterized by a post-treatment rise in total bilirubin, together with a decrease in C-reactive protein to approximately 5.3 mg/L and the C-reactive protein/albumin ratio to about 0.136. This hypothesis warrants prospective validation.

## 1. Introduction

Crohn’s disease(CD) is a chronic inflammatory disorder of the gastrointestinal tract from the mouth to the rectum with an unknown etiology [1–3]. The worldwide incidence of Crohn’s disease is continuously increasing [4,5]. A nationwide, population-based study in 2023 suggested that the incidence of Crohn’s disease in urban China in 2016 was 0.71 per 100,000 person-years (95% confidence interval,0.33–1.23) [6], with a steadily increasing trend. Patients with Crohn’s disease tend to undergo cycles of disease remission and relapse [7,8].This recurring pattern seriously impacts patients’ physical and mental health, leads to higher hospitalization rates and imposes a substantial burden on patients, their families, and society. Therefore early detection of disease activity is crucial to provide timely treatment and improve the prognosis as well as quality of life [9,10].Currently, endoscopy is regarded as the gold standard for disease diagnosis and assessment [11]. However, endoscopy is invasive, uncomfortable and expensive for patients, making it inconvenient for long-term monitoring of the disease [12,13]. Thus, this study aims to explore the role of serum biomarkers in the assessment of disease activity in Crohn’s disease patients with the objectives of reducing reliance on endoscopy, minimizing patient burden and healthcare costs, and enhancing long-term treatment adherence.

Bilirubin, an endogenous product of heme metabolism, has long been recognized for its antioxidative and anti-inflammatory properties [14,15].Several studies have demonstrated the correlation between reduced levels of total bilirubin and inflammatory bowel disease(IBD) [16–18].By comparing bilirubin levels between patients and healthy individuals, most studies have demonstrated that reduced bilirubin levels are associated with diminished antioxidant capacity and a consequently higher disease incidence. However, its potential role in assessing disease activity remains relatively unexplored. Uric acid, the final product of purine catabolism, is eliminated from the body with approximately two-thirds excreted by the kidneys in urine and one-third metabolized and excreted via the intestines. Qisheng Su et al. revealed that patients with Crohn’s disease had low uric acid, bilirubin and albumin compared with healthy controls [19]. On the contrary, Feng Zhu et al. illustrated that uric acid was significantly higher in patients with Crohn’s disease than in healthy controls, which was independent of disease activity, whereas uric acid/creatinine ratio(UA/Cr ratio) was associated with Crohn’s disease activity [1]. It remains elusive on uric acid levels and its potential significance in patients with Crohn’s disease. Serum albumin, generally regarded as an indicator of nutrition, also serves as a negative acute phase protein [20]. In recent years, growing research has shown that C-reactive protein/albumin ratio(CRP/ALB ratio) is associated with disease activity in patients with Crohn’s disease [8,21]. Most studies have assessed the CRP/ALB ratio at a single baseline time point, whereas few have explored its role in monitoring dynamic changes during post-treatment follow-up.

In this study, we first validate the established CRP/ALB ratio as a prognostic baseline and then extend our investigation to explore the potential independent role of total bilirubin and uric acid in assessing disease activity. By leveraging pre- and post-treatment measurements, we will further assess their dynamic changes and clinical applicability.

## 2. Materials and methods

### 2.1 Participants

In this retrospective single-center study, we included 170 CD patients who were hospitalized in the Second Hospital of Anhui Medical University (Hefei, China) between January 1, 2016, and July 31, 2023. All CD patients were newly diagnosed based on integrated clinical, laboratory, endoscopic, and histopathological criteria, aged ≥14 years, and had complete clinical data. A total of 100 age- and sex-matched controls were selected from the same hospital department during the study period. Control participants underwent elective endoscopy or a benign gastrointestinal consultation and met the following criteria: (1) endoscopic confirmation of absence of IBD and neoplasms; (2) age ≥ 14 years; and (3) availability of all essential clinical data. Exclusion criteria for both groups included active infection, malignancy, other systemic autoimmune diseases (except CD in the patient group), significant renal or hepatic dysfunction, significant heart disease, or missing key research data.

The study was approved by the hospital ethics committee with the approval number YX2023−180(F1). For adult participants, the written informed consent was signed by themselves; for minors, the written informed consent was signed by their parents or guardians. For some participants who were lost to follow-up, the requirement for consent was waived by the ethics committee.

### 2.2 Longitudinal cohort

For the longitudinal arm of the study, patients received standard-of-care treatment as clinically indicated. The therapeutic interventions included one or a combination of the following medication classes: corticosteroids such as prednisone, immunosuppressants such as azathioprine, biological agents such as infliximab, vedolizumab, or ustekinumab, and 5-aminosalicylic acid (5-ASA) preparations. The ‘before therapy’ assessment was conducted at diagnosis prior to treatment initiation, and the ‘after therapy’ assessment was conducted at 14–20 weeks, which corresponds to the standard timeframe for evaluating therapeutic response in clinical practice.

Of the 170 patients initially screened, 148 were diagnosed with active disease. Among these patients, 77 were included in the final longitudinal analysis. The primary reasons for exclusion were loss to follow-up, for instance due to patients failing to return for scheduled visits or seeking subsequent care at other institutions, and incomplete data resulting from missing essential laboratory or clinical parameters needed for the study outcomes. We acknowledge that this non-random exclusion may introduce selection bias, as the included patients likely had better treatment adherence.

### 2.3 Data collection

Clinical and laboratory parameters were retrieved from the database of electronic medical record system in the hospital during a dedicated data collection period from December 23, 2023, to January 15, 2024. The extracted data included demographic characteristics (gender, age, body mass index, smoking status), disease features (age at diagnosis, localization, behavior), and key laboratory values (CRP, albumin, total bilirubin, uric acid, and creatinine). Written informed consent was obtained from the majority of participants. For the remaining participants who were lost to follow-up, the requirement for informed consent was formally waived by the Ethics Committee. All data were fully anonymized prior to analysis and the authors had no access to any information that could identify individual participants during or after data collection.

According to the Montreal classification system, the disease phenotype of all Crohn’s disease patients was defined by three parameters: age at diagnosis, categorized as A1 (≤16 years), A2 (17–40 years), or A3 (>40 years); disease location, designated as L1 (terminal ileum), L2 (colon), or L3 (ileocolon), with L4 as a modifier for isolated upper gastrointestinal disease that can be added to L1-L3; and disease behavior, classified as B1 (non-stricturing, non-penetrating), B2 (stricturing), or B3 (penetrating), with ‘p’ as a modifier for concomitant perianal disease.

Disease activity status was assigned based on a hierarchical approach. For the majority of patients with complete data, the Crohn’s Disease Activity Index (CDAI) was the primary criterion, with a score ≥150 defining active disease and <150 defining remission. For the small subset of patients lacking CDAI data, endoscopic findings served as the alternative criterion, where the presence of ulcers or significant erosions defined active disease, and their absence defined endoscopic remission.

### 2.4 Biochemical analysis

Serum biochemical biomarkers, including total bilirubin, albumin, uric acid, and creatinine, were measured using a Beckman Coulter AU5800 automated clinical chemistry analyzer. The detection principles and reference ranges were as follows: total bilirubin was measured by the diazonium salt method (reference range: 0–21 μmol/L for females and 0–26 μmol/L for males), albumin by the bromocresol green method (40–55 g/L), uric acid by the uricase-peroxidase method (155–357 μmol/L for females and 208–428 μmol/L for males), and creatinine by the Jaffe kinetic method (41–73 μmol/L for females and 57–97 μmol/L for males). CRP was measured using a Mindray BC7500 automated analyzer via an immunoturbidimetric assay (reference: < 6.0 mg/L). All analyses were performed in the central laboratory of our institution, a tertiary care hospital in China. The laboratory operates under a comprehensive quality management system, including daily calibration, internal quality control (IQC), and participation in external quality assessment (EQA) programs, which ensures the reliability and consistency of all results.

### 2.5 Statistical analysis

Normally distributed data were represented as the means ± standard deviation and data with skewed distribution as the median (interquartile range [Q1-Q3]). Categorical variables were expressed as number (percentage). Student *t* test was used for comparison of normally distributed variables between two groups, while ANOVA for comparison between multiple groups. Non-parametric rank sum test was carried out for non-normally distributed variables. χ2 test or Fisher’s exact test was conducted to analyze categorical variables. Spearman’s correlation was performed to determine the relationship of relevant parameters with Crohn’s disease activity.

A multivariate logistic regression model was constructed to identify factors independently associated with disease activity (CDAI ≥150), adjusting for age, sex, disease duration, and smoking status. Body mass index (BMI) was excluded from the model as it was conceptualized as a concomitant outcome of the disease process rather than a true confounder. To avoid potential multicollinearity between derived ratios and their component variables, the model incorporated the five original biochemical variables (bilirubin, albumin, CRP, uric acid, creatinine) rather than composite ratio indices, thereby allowing for direct interpretation of each variable’s independent effect. To further assess multicollinearity among these correlated biomarkers, the variance inflation factor (VIF) was calculated for each variable in the model, with a VIF value of less than 5 indicating no severe multicollinearity.

The discriminatory power of each biomarker was assessed using receiver operating characteristic (ROC) curve analysis. The area under the curve (AUC) was calculated, and its statistical significance (tested against the null hypothesis of AUC = 0.5) was determined by a Z-test. The optimal cut-off value was identified by maximizing Youden’s index and reported along with its corresponding sensitivity and specificity. All statistical tests were carried out using the SPSS statistical software (version 26.0), and a two-sided *P* value of < 0.05 was considered statistically significant.

## 3. Results

A total of 270 individuals were included in this study, comprising 170 CD patients (45 women and 125 men) and 100 healthy controls (28 women and 72 men). The median age of the CD patients was 27 years old (IQR 21–35.25), and for the healthy controls, it was 28 years old (IQR 21–39). Among the CD patients,22 patients were classified as having inactive disease, while 148 patients were classified as having active disease. Of the active group,80 patients were further divided into the mild disease activity group, and 68 patients were assigned to the moderate-to-severe disease activity group. The demographic and clinical characteristics of the study cohort (CD patients and healthy controls) were summarized in Table 1. No significant differences were observed in age, gender or smoking history between the CD and healthy control groups.

**Table 1:** Baseline characteristics of the study cohort.

Characteristics	CD patients (n = 170)	Healthy controls (n = 100)
Age(years)	27.00(21.00, 35.25)	28.00(21.00, 39.00)
Sex(male/female)	45/125	28/72
Smoking(yes/no)	10/160	6/87
BMI(Kg/m^2^)	19.58(17.47, 21.48)	21.94(19.92, 24.96)
Age at diagnosis, year		
A1(≤16 years)	18(10.59%)	–
A2(17–40 years)	123(72.35%)	–
A3(>40 years)	29(17.06%)	–
**Disease location**		
L1 (terminal ileum)	28(16.47%)	–
L2(colon)	27(15.88%)	–
L3 (ileocolon)	115(67.65%)	–
**Disease behavior**		
**B1(non-stricturing, non-penetrating)**	110(64.71%)	–
B2(stricturing)	28(16.47%)	–
B3(penetrating)	25(14.71%)	–
B2 + B3	7(4.11%)	–
Perianal disease		
Yes	115(67.65%)	–
No	55(32.35%)	–
Clinical disease activity		
Inactive group	22(12.94%)	–
Mild group	80(47.06%)	–
Moderate-to-severe group	68(40.00%)	–

Values are expressed as n (%) or median (Interquartile range);

CD, Crohn’s disease; BMI, body mass index;

B2 + B3, patients with both stricturing and penetrating disease.

As compared to the healthy controls, CD patients exhibited significantly lower total bilirubin and albumin levels, alongside significantly elevated CRP levels and a higher CRP/ALB ratio. The differences, 95% confidence intervals (95%CI), and *P* values for these biomarkers are presented in Table 2. In contrast, no significant differences were observed in uric acid, creatinine, or the UA/Cr ratio.

**Table 2:** Comparison of biomarker levels between Crohn’s disease patients and healthy controls.

Biomarkers	CD patients	Healthy controls	Difference(95% CI)	*P*
Bilirubin(μmol/L)	8.75(6.68, 11.50)	12.65(9.30, 15.78)	−3.40(−4.40, −2.40)	< 0.001
Uric acid(μmol/L)	314.00(249.75, 378.50)	340.50(291.50, 376.75)	−18.00(−38.00, 3.00)	0.086
Creatinine (μmol/L)	63.00(52.00, 75.00)	64.50(55.50, 72.75)	−1.00(−4.00, 3.00)	0.698
UA/Cr ratio	5.09(3.86, 6.48)	5.18(4.68, 5.88)	−0.10(−0.47, 0.28)	0.613
CRP(mg/L)	19.65(5.00, 50.18)	0.50(0.50, 1.20)	18.80(13.40, 29.40)	< 0.001
Albumin(g/L)	34.88 ± 6.60	42.74 ± 2.54	−7.86(−8.98, −6.75)	< 0.001
CRP/ALB ratio	0.58(0.13, 1.65)	0.01(0.01, 0.03)	0.56(0.40, 0.99)	< 0.001

Values are expressed as mean±standard deviation or median (Interquartile range);

Difference is presented as Mean Difference for normally distributed data (Albumin) and as Median Difference (Hodges-Lehmann estimate) for non-normally distributed data (all other biomarkers),

calculated as (“CD patients” – “Healthy controls”); A positive value indicates that the level is higher in CD patients than in healthy controls.

CD, Crohn’s disease; CI, confidence interval; UA/Cr ratio = uric acid/creatinine ratio;

CRP, C-reactive protein; ALB, albumin; CRP/ALB ratio = C-reactive protein/albumin ratio.

As shown in Table 3, CD patients were stratified into three groups based on their CDAI scores: inactive, mild, and moderate-to-severe. One-way ANOVA or Kruskal-Wallis tests revealed significant differences across these groups in total bilirubin, albumin, CRP, and the CRP/ALB ratio, but not in uric acid, creatinine, or the UA/Cr ratio (see Table 4 for post-hoc pairwise comparisons). Specifically, albumin levels decreased significantly with increasing disease activity, whereas CRP and the CRP/ALB ratio showed a significant increasing trend (all pairwise comparisons, *P* < 0.001). In contrast, total bilirubin demonstrated a distinct pattern, with a significant difference observed solely between the inactive and moderate-to-Severe groups (*P* = 0.041).

**Table 3:** Comparison of biomarker levels by disease activity in Crohn’s disease.

Biomarkers	Inactive group	Mild group	Moderate-to-severe group	*P*
Bilirubin(μmol/L)	10.85(7.68, 13.83)	8.75(6.63, 11.50)	8.05(6.53, 10.15)	0.046
Uric acid(μmol/L)	323.00(258.75, 437.00)	325.00(253.75, 380.00)	302.50(236.25, 369.25)	0.122
Creatinine (μmol/L)	62.00(53.00, 75.25)	64.50(53.00, 75.00)	62.50(50.25, 76.00)	0.948
UA/Cr ratio	5.72(4.27, 7.25)	5.16(3.86, 6.56)	4.94(3.86, 6.02)	0.257
CRP(mg/L)	0.65(0.50, 3.55)	14.60(5.25, 37.30)	45.20(20.78, 70.00)	< 0.001
Albumin(g/L)	42.48 ± 3.53	35.54 ± 5.96	31.64 ± 5.83	< 0.001
CRP/ALB ratio	0.02(0.01, 0.08)	0.43(0.15, 1.17)	1.49(0.58, 2.38)	< 0.001

Values are expressed as mean±standard deviation or median (Interquartile range);

UA/Cr ratio = uric acid/creatinine ratio; CRP, C-reactive protein; ALB, albumin;

CRP/ALB ratio= C-reactive protein/albumin ratio.

**Table 4:** Pairwise comparisons of biomarker levels across disease activity groups in Crohn’s disease.

Biomarkers	Group Comparison	Difference (95% CI)	*P*
Bilirubin(μmol/L)	Inactive vs. Mild	1.50(−0.30,3.20)	0.247
	Inactive vs. Moderate-to-Severe	2.20(0.50, 4.00)	0.041
	Mild vs. Moderate-to-Severe	0.60(−0.40, 1.70)	0.768
CRP(mg/L)	Inactive vs. Mild	−12.00(−21.40, −6.60)	< 0.001
	Inactive vs. Moderate-to-Severe	−40.25(−53.20, −32.90)	< 0.001
	Mild vs. Moderate-to-Severe	−24.50(−33.50, −13.20)	< 0.001
Albumin(g/L)	Inactive vs. Mild	6.94(3.72, 10.16)	< 0.001
	Inactive vs. Moderate-to-Severe	10.84(7.56, 14.12)	< 0.001
	Mild vs. Moderate-to-Severe	3.90(1.69, 6.10)	< 0.001
CRP/ALB ratio	Inactive vs. Mild	−0.37(−0.61, −0.19)	< 0.001
	Inactive vs. Moderate-to-Severe	−1.35(−1.80, −1.05)	< 0.001
	Mild vs. Moderate-to-Severe	−0.85(−1.16, −0.46)	< 0.001

Difference for Albumin is reported as mean difference based on Tukey HSD post hoc test following one-way ANOVA**.** Differences for Bilirubin, CRP, and CRP/ALB ratio are reported as median differences based on Dunn’s post hoc test following Kruskal-Wallis test, with Bonferroni correction for multiple comparisons;

For all pairwise comparisons, the difference is calculated as (First group – Second group), following the order presented. A positive value indicates a higher value in the first group;

CI, confidence interval; CRP, C-reactive protein; ALB, albumin;

CRP/ALB ratio= C-reactive protein/albumin ratio.

A longitudinal analysis of 77 patients with active CD was conducted to assess biomarker dynamics. Following therapy, patients were stratified into a remission group (n = 51) and an active group (n = 26) based on treatment response. When comparing pre-and post-treatment levels within each group, both the remission and active groups showed a significant increase in albumin and a significant decrease in CRP and the CRP/ALB ratio after treatment. Notably, a significant rise in total bilirubin was exclusive to the remission group. No significant dynamic changes were observed for uric acid, creatinine, or the UA/Cr ratio in either cohort (Tables 5 and 6). The comparisons between the remission and active groups revealed significant pre-existing differences: prior to treatment, the active group demonstrated higher CRP levels and a higher CRP/ALB ratio than the remission group, whereas albumin and total bilirubin levels were comparable. Following treatment, the remission group showed significantly higher albumin and total bilirubin levels, together with lower CRP levels and CRP/ALB ratio, compared to the active group.

**Table 5:** Dynamic changes of biomarkers in the remission group following treatment.

Biomarkers	pre-treatment	post-treatment	Difference (95% CI)	*P*
Bilirubin(μmol/L)	8.90(6.60, 11.50)	11.50(8.90, 15.00)^△^	2.65(1.10, 3.90)	0.001
Uric acid(μmol/L)	319.00(255.00, 378.00)	322.00(279.00, 374.00)	9.25(−16.00, 26.50)	0.383
Creatinine (μmol/L)	61.00(50.00, 71.00)	65.00(52.00, 4.00)	2.50(−1.50, 6.00)	0.239
UA/Cr ratio	5.63(4.13, 6.94)	5.28(4.60, 6.09)	−0.18(−0.67, 0.25)	0.426
CRP(mg/L)	20.20(8.30, 46.70)^*^	1.00(0.50, 4.70)^△^	−24.05(−32.10, −17.30)	< 0.001
Albumin(g/L)	34.72 ± 6.13	41.73 ± 4.17^△^	7.01(5.43, 8.59)	< 0.001
CRP/ALB ratio	0.61(0.20, 1.54)^*^	0.02(0.01, 0.10)^△^	−0.79(−1.04, −0.53)	< 0.001

Values are expressed as mean±standard deviation or median (Interquartile range);

Difference is presented as Mean Difference for normally distributed data (Albumin) and as Median Difference (Hodges-Lehmann estimate) for non-normally distributed data (all other biomarkers), calculated as (post-treatment – pre-treatment). A positive value indicates an increase from pre-treatment;

CI, confidence interval; UA/Cr ratio=uric acid/creatinine ratio;

CRP, C-reactive protein; ALB, albumin; CRP/ALB ratio = C-reactive protein/albumin ratio;

* *P* < 0.05 compared to active group in pre-treatment;

^△^*P* < 0.05 compared to active group in post-treatment.

**Table 6:** Dynamic changes of biomarkers in the active group following treatment.

Biomarkers	pre-treatment	post-treatment	Difference (95% CI)	*P*
Bilirubin(μmol/L)	8.65(7.08, 10.15)	9.60(7.08, 12.95)	0.80(-0.85, 3.00)	0.298
Uric acid(μmol/L)	325.00(264.25, 390.75)	352.50(249.00, 389.25)	13.00(-22.50, 49.00)	0.439
Creatinine (μmol/L)	60.50(53.75, 75.75)	64.00(52.75, 72.25)	0.00(-6.00, 4.50)	0.943
UA/Cr ratio	4.99(4.16, 6.57)	5.31(4.62, 5.98)	0.16(-0.37, 0.72)	0.603
CRP(mg/L)	50.05(16.25, 73.33)	19.05(8.13, 34.88)	-21.55(-39.05, -1.65)	0.034
Albumin(g/L)	33.22 ± 5.82	38.50 ± 4.61	5.28(2.43, 8.12)	0.001
CRP/ALB ratio	1.60(0.42, 2.30)	0.49(0.22, 0.89)	-0.82(-1.34, -0.16)	0.006

Values are expressed as mean±standard deviation or median (Interquartile range);

Difference is presented as Mean Difference for normally distributed data (Albumin) and as Median Difference (Hodges-Lehmann estimate) for non-normally distributed data (all other biomarkers), calculated as (post-treatment – pre-treatment). A positive value indicates an increase from pre-treatment;

CI, confidence interval; UA/Cr ratio=uric acid/creatinine ratio;

CRP, C-reactive protein; ALB, albumin; CRP/ALB ratio = C-reactive protein/albumin ratio.

As shown in Table 7, Spearman correlation analysis demonstrated moderate associations of CD activity with CRP (r = 0.548, *P* < 0.001), the CRP/ALB ratio (r = 0.570, *P* < 0.001), and albumin (r = −0.503, *P* < 0.001). In contrast, weak associations were observed with total bilirubin (r = −0.176, *P* = 0.022) and uric acid (r = −0.158, *P* = 0.04). No significant associations were detected for creatinine or the UA/Cr ratio.

**Table 7:** Spearman correlations of biomarkers with disease activity in Crohn’s disease.

Biomarkers	r_s_	*P*
Bilirubin	−0.176	0.022
Uric acid	−0.158	0.040
Creatinine	−0.022	0.780
UA/Cr ratio	−0.122	0.113
CRP	0.548	<0.001
Albumin	−0.503	<0.001
CRP/ALB ratio	0.570	<0.001

UA/Cr ratio = uric acid/creatinine ratio; CRP, C-reactive protein; ALB, albumin;

CRP/ALB ratio= C-reactive protein/albumin ratio.

The multivariate logistic regression model identified several independent factors associated with disease activity (CDAI ≥150). The model confirmed the absence of severe multicollinearity, with all VIF values well below the threshold of 5 (albumin: 1.59; CRP: 1.35; uric acid: 1.34; total bilirubin: 1.22; creatinine: 1.16), indicating that multicollinearity did not pose a concern for model stability. After adjustment for age, sex, disease duration and smoking status, higher CRP levels (OR = 1.142, 95% CI: 1.053–1.238, *P* = 0.001) and lower albumin levels (OR = 0.716, 95% CI: 0.625–0.821, *P* < 0.001) remained significantly associated with active CD. Total bilirubin was also identified as an independent protective factor (OR = 0.874, 95% CI: 0.770–0.992, *P* = 0.037). In contrast, neither uric acid (OR = 0.995, *P* = 0.073) nor creatinine (OR = 0.998, *P* = 0.903) showed statistically significant associations with disease activity in the final model. The detailed results are presented in Table 8.

**Table 8:** Multivariate logistic regression analysis for predicting active Crohn’s disease.

Biomarkers	B	SE	Wald	*P*	OR	95%CI
Bilirubin	−0.135	0.064	4.363	0.037	0.874	0.770-0.992
Uric acid	−0.005	0.003	3.207	0.073	0.995	0.990-1.000
Creatinine	−0.002	0.015	0.015	0.903	0.998	0.969-1.028
CRP	0.133	0.041	10.345	0.001	1.142	1.053-1.238
Albumin	−0.334	0.070	23.044	<0.001	0.716	0.625-0.821

The OR (odds ratios) were adjusted for age, sex, disease duration and smoking status;

CI, confidence interval; CRP, C-reactive protein.

**Fig 1:**
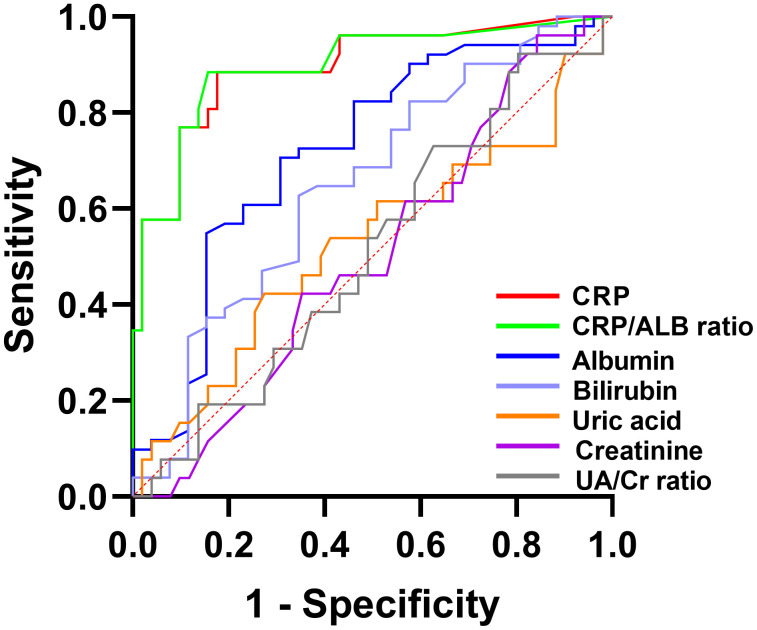
Receiver operating characteristic (ROC) curve of biomarkers for predicting active Crohn’s disease. CRP, C-reactive protein; ALB, albumin; CRP/ALB ratio = C-reactive protein/albumin ratio; UA/Cr ratio = uric acid/creatinine ratio.

The predictive performance of the biomarkers was evaluated using ROC curve analysis. The discriminatory power of each biomarker is summarized in Table 9, with a visual representation provided in Fig 1. The analysis revealed that the CRP/ALB ratio demonstrated excellent discriminatory power (AUC = 0.903), followed by CRP alone (AUC = 0.894). Albumin showed acceptable discriminatory ability (AUC = 0.719). In contrast, total bilirubin exhibited only weak, albeit above-chance discriminatory ability (AUC = 0.648). Other markers, including uric acid, creatinine, and the UA/Cr ratio, showed no significant predictive value, as their AUCs were not statistically significant (*P* > 0.05).

**Table 9:** Discriminatory power of biomarkers for Crohn’s disease activity based on ROC analysis.

Biomarkers	AUC	95%CI	*P*	Cut-offs	Youden’s index	Sensitivity(%)	Specificity(%)
Bilirubin	0.648	0.514-0.782	0.035	10.45	0.281	62.7	65.4
Uric acid	0.528	0.384-0.672	0.686	–	–	–	–
Creatinine	0.503	0.370-0.635	0.970	–	–	–	–
UA/Cr ratio	0.512	0.377-0.647	0.863	–	–	–	–
CRP	0.894	0.815-0.973	<0.001	5.30	0.708	88.5	82.4
Albumin	0.719	0.593-0.846	0.002	39.95	0.398	70.6	69.2
CRP/ALB ratio	0.903	0.832-0.975	<0.001	0.136	0.728	88.5	84.3

ROC, receiver operating characteristic; AUC, area under the curve; CI, confidence interval;

Youden’s index (J = sensitivity + specificity – 1); UA/Cr ratio = uric acid/creatinine ratio;

CRP, C-reactive protein; ALB, albumin; CRP/ALB ratio = C-reactive protein/albumin ratio.

## 4. Discussion

Crohn’s disease is a relapsing-remitting inflammatory disease of the digestive system, requiring long-term disease activity and severity assessment [22]. Therefore, it is of great value to search convenient and effective biomarkers for clinical practice. In this study we found that total bilirubin and albumin levels were lower in CD patients than those in healthy controls, while CRP and the CRP/ALB ratio were higher. Additionally, CRP and the CRP/ALB ratio were positively associated with CD disease activity, whereas total bilirubin and albumin were negatively correlated with CD disease activity. With the exception of a single weak correlation in Spearman’s analysis, uric acid showed no significant associations with disease activity overall.

Oxidative stress and antioxidant deficiency are considered to play key roles in the pathogenesis of CD [23–25]. Mounting evidence indicated that bilirubin exerted important antioxidant activity against oxidative damage [15,26,27]. Lenicek et al demonstrated that each 1 mmol/L decrease in serum bilirubin was related to a 13% increase in the risk of CD manifestation [16]. Consistent with this, a meta-analysis by Zoroddu et al revealed that bilirubin levels were generally lower in patients with IBD than in healthy controls [27]—an observation that our study in CD directly confirms. While the observation of reduced bilirubin in Crohn’s disease is well-established, the underlying pathophysiological mechanisms remain incompletely defined. Moving beyond its conventional role as an antioxidant, bilirubin is now recognized as a signaling molecule that activates the Nuclear factor erythroid 2-related factor 2/Heme oxygenase-1 (Nrf2/HO-1) pathway [28]. Notably, studies have shown that Nrf2 activation not only upregulates the transcription of antioxidant genes but also directly inhibits the expression of reactive oxygen species(ROS) -mediated pro-inflammatory genes, such as COX2 [29], underscoring the dual protective function of this pathway. Given that the Nrf2/HO-1 axis is a promising therapeutic target for IBD—its activation counteracts oxidative stress, regulates intestinal flora, and repairs the mucosal barrier [30] —we hypothesize that hypobilirubinemia in CD results in inadequate activation of this cytoprotective system. This insufficiency may have profound implications within the chronic inflammatory milieu of CD. In this setting, oxidative stress and aberrantly activated inflammatory signaling pathways such as the JAK/STAT pathway can form a self-perpetuating vicious cycle [31]. Consequently, we speculate that bilirubin deficiency in CD likely impairs the Nrf2-mediated dual defense system—antioxidant and anti-inflammatory—thereby disrupting intestinal mucosal homeostasis and ultimately exacerbating inflammation and tissue injury. Our study not only confirmed reduced total bilirubin levels in CD patients, but more significantly, logistic regression analysis identified total bilirubin as an independent predictor of disease activity (OR = 0.874, 95% CI: 0.770–0.992, *P* = 0.037). Furthermore, ROC analysis yielded an AUC of 0.648, indicating that total bilirubin possess modest but independent predictive value, although its AUC value was lower than those of CRP and the CRP/ALB ratio. Notably, longitudinal assessment of 77 active CD patients revealed a distinct pattern: a significant rise in total bilirubin was exclusive to the remission group, while CRP, albumin and the CRP/ALB ratio showed remarkable alterations in both remission and active groups. Consequently, we propose that an absolute increase in serum total bilirubin following treatment may serve as a valuable complementary marker for predicting serological remission in CD.

Serum albumin, primarily synthesized in the liver, is a well-established marker of nutritional status in chronic diseases [32]. However, its role extends beyond nutrition, as it is also recognized as a negative acute-phase protein whose synthesis is influenced during systemic inflammatory responses [20,33]. Accordingly, albumin has been regarded as a reliable indicator of disease severity in inflammatory conditions [34]. In Crohn’s disease, serum albumin levels are influenced by a broader spectrum of pathophysiological factors beyond nutritional status and systemic inflammation. For instance, diarrhea—a common clinical manifestation—may lead to hemoconcentration, resulting in a relative elevation of albumin concentration. Conversely, intestinal inflammation causes protein-losing enteropathy due to loss of gut barrier integrity [35]. It should also be noted that, while patients with overt liver dysfunction were excluded from this study, hepatic synthesis function remains a major determinant of serum albumin levels in the broader Crohn’s disease population. Thus, despite the observed inverse correlation with disease activity, the multifactorial nature of albumin underscores that this correlation is non-specific, and these confounders must be considered when interpreting its clinical significance.

CRP, known as a positive acute phase protein, is primarily produced in the liver and stimulated by tumor necrosis factor α(TNF-α),interleukin-6(IL-6)and interleukin-1(IL-1) [36,37].Some studies have shown that CRP is suitable for long-term monitoring of Crohn’s disease [38,39].However, CRP levels can also be affected by other physiological conditions such as bacterial and viral infections. Recently, an increasing number of reports have suggested that the CRP/ALB ratio is useful for identifying disease activity in patients with CD [8,21,40,41]. Our results strongly corroborate this finding, as the CRP/ALB ratio demonstrated excellent discriminatory performance, with an AUC of 0.903—surpassing that of CRP alone (AUC = 0.894), albumin alone (AUC = 0.719), and total bilirubin (AUC = 0.648). In the longitudinal cohort, a further comparison between the remission and active groups revealed that, prior to treatment, patients in the active group had significantly higher CRP levels and a higher CRP/ ALB ratio, whereas albumin and total bilirubin levels were comparable. Based on these findings, we speculate that the CRP/ ALB ratio and CRP may possess predictive value for assessing therapeutic response.

Studies by Qisheng Su et al. [19] and Feng Zhu et al. [1] have reported contradictory conclusions regarding uric acid levels. In our study, Spearman’s analysis revealed a weak correlation. However, no other significant differences were detected. To reconcile these disparate findings, we propose a “phase-dependent dual-role” framework to explain uric acid’s varying roles in different disease stages. In acute/severe inflammation, tissue necrosis may release uric acid as a potential endogenous danger signal that could activate the NLRP3 inflammasome pathway [42], providing a plausible explanation for the positive correlation reported by Feng Zhu et al. [1] in their acute-phase patient cohort. Conversely, during chronic/mild inflammation, long-term inflammatory consumption and malnutrition appear to reduce uric acid precursor availability, while persistent oxidative stress seems to deplete uric acid through its antioxidant functions [43], collectively suggesting a possible “exhaustion state” that aligns with the negative correlation observed by Qisheng Su et al. [19] in their chronic-phase patient cohort. Our data indicated a non-significant trend of decreasing uric acid levels (healthy controls > patients in remission > active disease patients) that corresponds with the weak Spearman correlation. Furthermore, our active-disease cohort predominantly had mild activity, while the number of patients with moderate-to-severe, and especially severe disease was relatively small. The exhaustion mechanism likely predominated but was counterbalanced by acute-phase effects in a minority of patients, ultimately accounting for the overall lack of statistical significance.

Based on the characteristic pattern of biomarkers observed in active CD patients—specifically decreased total bilirubin and albumin, elevated CRP and the CRP/ALB ratio, and a non-significant trend toward lower uric acid—we propose an integrated conceptual framework that positions these markers within the core pathophysiological axis of CD: “oxidative stress–inflammation–barrier injury”.Within this model, decreased bilirubin signals upstream depletion of antioxidant reserves and impaired activation of the Nrf2/HO-1 pathway [29,30]. The ensuing systemic inflammatory burst is captured by CRP, which directly reflects inflammatory intensity, and the CRP/ALB ratio, which integrates the inflammatory load with nutritional and synthetic reserve consumption. Reduced albumin represents the convergence of multiple pathways: inflammation-mediated suppression of hepatic synthesis, protein-losing enteropathy [35], and chronic consumption. The potential phase-dependent role of uric acid further underscores the dynamic interplay between oxidative stress and inflammation. Collectively, this coordinated biomarker shift—weakened antioxidant defense, amplified systemic inflammation, and depleted physiological reserves—maps the disease continuum from initial redox imbalance to systemic compromise in CD.

To translate these findings into clinical practice, we further propose a **“**Multi-biomarker serological remission model.” This model hypothesizes that effective treatment may be indicated by a combined improvement in key serum markers: an increase in total bilirubin (based on observed associations in Table 5 and 6), along with a reduction in CRP to around 5.3 mg/L and the CRP/ALB ratio to roughly 0.136 (cutoff values derived from ROC analysis), accompanied by the alleviation of clinical symptoms. This integrated approach may offer a potential framework for defining and monitoring serological remission in CD. The validity and clinical utility of this model require prospective validation.

Derived from the model, we propose a personalized surveillance approach for Crohn’s disease patients. For those predicted to be in remission, the routinely mandatory first endoscopic assessment at 14–20 weeks could potentially be avoided. Furthermore, for patients who maintain sustained serological remission, the endoscopy interval could be extended from the standard annual schedule to once every two years. We estimate that this two-tiered strategy could reduce the long-term endoscopic frequency by approximately 50%. This reduction would not only alleviate patient discomfort and procedural risks but also translate into substantial cost savings for the healthcare system, for instance, considering the representative cost of a painless colonoscopy, which is approximately 800 RMB per procedure. It is crucial to emphasize, however, that these estimates are preliminary and intended to illustrate potential benefits; their validity and implementation require formal prospective validation in future studies designed to evaluate such biomarker-guided management pathways.

Our study had several potential limitations. First, as a single-center retrospective study with a relatively small sample size, it is susceptible to selection bias. Second, we did not account for other factors, such as medications and diet, which may have influenced our findings. Third, disease activity and related biomarkers were only analyzed at two discrete time points, with no follow-up, preventing us from observing changes in biomarkers and disease activity over time. Therefore, a prospective, multi-center study with a larger sample size is necessary to further validate our results.

## 5. Conclusion

In conclusion, this study delineates a distinct serum biomarker profile in Crohn’s disease, characterized by elevated CRP and CRP/ALB ratio alongside decreased total bilirubin and albumin. The observation of only a limited, weak correlation for uric acid underscores the complexity of its role. Building on the ROC-derived cut-off values and longitudinal assessment, we propose a “Multi-biomarker serological remission model”. This model is defined by a composite post-treatment shift: an increase in total bilirubin concurrent with reductions in CRP (to around 5.3 mg/L) and the CRP/ALB (to around 0.136). For patients meeting this biochemical profile, endoscopic confirmation could be strategically deferred, potentially reducing the frequency of invasive procedures in long-term management. This two-tiered strategy could alleviate patient burden and optimize healthcare resource utilization. Given the retrospective, single-center design of this study, prospective multicenter validation is essential to confirm the clinical utility of this model and to refine the proposed thresholds.

## Supporting information

S1 File Data-English. (ZIP)

## Abbreviations

CD: Crohn’s disease
UC: ulcerative colitis
IBD: inflammatory bowel disease
CRP: C-reactive protein
ALB: albumin
CDAI: Crohn’s Disease Activity Index
CRP/ALB ratio: C-reactive protein/albumin ratio
BMI: Body mass index
UA/Cr ratio: uric acid/creatinine ratio
CI: confidence interval
ROC: receiver operating characteristic
AUC: under the curve
VIF: variance inflation factor.
